# CD14^+^ Cells with the Phenotype of Infiltrated Monocytes Consist of Distinct Populations Characterized by Anti-inflammatory as well as Pro-inflammatory Activity in Gouty Arthritis

**DOI:** 10.3389/fimmu.2017.01260

**Published:** 2017-10-06

**Authors:** Ji Hye Jeong, Seokchan Hong, Oh Chan Kwon, Byeongzu Ghang, Inseok Hwang, Yong-Gil Kim, Chang-Keun Lee, Bin Yoo

**Affiliations:** ^1^Division of Rheumatology, Department of Internal Medicine, University of Ulsan College of Medicine, Asan Medical Center, Seoul, South Korea; ^2^Asan Institute for Life Science, Asan Medical Center, Seoul, South Korea

**Keywords:** gout, monocyte, macrophage, inflammation, phagocytosis

## Abstract

It has been suggested that inflammasome-mediated IL-1β production in monocytic cells is responsible for the acute inflammatory response in gouty arthritis. However, phenotypical and functional analyses of monocytes during gouty arthritis have yet to be conducted. Therefore, we investigated the characteristics of monocytes/macrophages in the synovial fluid cells of patients with acute gout. The number and frequency of monocytes/macrophages in the synovial fluid mononuclear cells (SFMCs) of patients was examined. The expression of markers for monocyte recruitment and tissue-resident macrophages, the production of pro-inflammatory and anti-inflammatory cytokines, and phagocytosis were analyzed in the monocytes/macrophages of patients with acute gout attacks. The number and frequency of CD14^+^CD3^−^CD19^−^CD56^−^ monocytes/macrophages was markedly increased in the SFMCs of patients with gout compared to those of patients with rheumatoid arthritis (RA). CD14^+^ cells showed the phenotypes of infiltrated monocytes rather than tissue-resident macrophages, characterized by a high expression of CCR2, MRP8, and MRP14, but a low expression of MERTK and 25F9. These cells had the capacity to produce pro-inflammatory cytokines such as TNF-α and IL-1β after stimulation with lipopolysaccharides. In addition, anti-inflammatory features, including CD163 expression and IL-10 production from CD14^+^ cells, were significantly higher in patients with gout than in those with RA. CD14^+^ cells with phenotype of M2 macrophages had high phagocytic activity for monosodium urate crystals. Thus, our results indicate that monocytes/macrophages from patients with gout have the phenotype of infiltrated monocytes, and these cells consist of different populations characterized by anti-inflammatory activities as well as pro-inflammatory functions.

## Introduction

Gout is a common, debilitating inflammatory arthritis caused by the deposition of monosodium urate (MSU) crystals. Acute gout attacks are characterized by abrupt, marked painful arthritis but resolve spontaneously within a week (1, 2). Inflammation in acute gout attacks is characterized by the production of various pro-inflammatory cytokines such as IL-1β, IL8, and TNF-α, and the infiltration of neutrophils (3–5).

Recent works have demonstrated that monocytes/macrophages play a key role in MSU-driven pro-inflammatory cytokine production during the initiation of the acute inflammatory response (6–8). In particular, a previous report showed that resident macrophages are likely to be crucial during the early inflammatory response in a murine MSU-peritonitis model (6). It has been known for a long time that tissue-residing macrophages originate from circulating monocytes, which are recruited into tissues and mature to macrophages (9). Recent studies, however, showed that peripheral tissues have a specific tissue-resident macrophage population that arises from precursors during embryogenesis. These tissue-resident macrophages are maintained by local self-renewal without significant contribution from circulating monocytes in the peripheral blood (10, 11). On the other hand, circulating monocytes can migrate, differentiate into macrophages, and become involved in the pathogenesis of various acute and chronic inflammatory diseases (12, 13). Although infiltrated monocytes can be distinguished from tissue-resident macrophages according to the expression of myeloid-related protein 8 (MRP8), MRP14, and CCR2, tissue-resident macrophages are identified by MERTK and 25F9 expression (14–16). The recruitment of circulating monocytes is dependent on the arrangement of various adhesion molecules, chemokines, and complement (13, 17, 18). Actually, it has been reported that C5a and C5a receptor signaling is important for leukocyte infiltration in the MSU-induced peritonitis model (19).

Monocytes/macrophages are potent phagocytic cells that play a role in the first line of defense against invading pathogens and danger signals. Phagocytosis of MSU crystals by macrophages have been considered to be crucial for the resolution of MSU-mediated acute inflammation (20, 21). However, recent studies have shown that phagocytosis of MSU crystals is associated with initiation of inflammation. Internalized MSU crystals can damage lysosomal membranes, thereby leading to phagolysosome rupture and activation of the NLRP3 inflammasome signaling (22, 23). It is responsible for the activation of IL-1β and IL-18 in gouty inflammation.

Further, macrophages have various heterogeneities in their phenotypes and function according to the anatomical location and disease condition. It is well established that there are two distinct subsets within the macrophage population known as M1, i.e., pro-inflammatory macrophages, and M2, i.e., anti-inflammatory macrophages (24, 25). M1 macrophages secrete pro-inflammatory cytokines such as TNF-α, IL-6, IL-1β, and IL-12. In contrast, M2 macrophages are considered anti-inflammatory and immune-regulatory because they produce IL-10 and TGF-β. In addition, M2 macrophages show prominent phagocytic activity that can be promoted by IL-10 (26, 27).

Previously, although the role of monocytes/macrophages was described in MSU-mediated inflammation in a murine model (6, 28), phenotypical and functional analyses, including a phagocytosis of MSU crystals, of monocytes/macrophages in human patients with gout have not been performed thus far. Furthermore, little is known about the mechanisms involved in the recruitment of circulating monocytes during acute gout attacks. Therefore, in the present study, we investigated the phenotypical and functional characteristics of synovial monocytes/macrophages in gout patients with acute attacks.

## Materials and Methods

### Study Subjects and Synovial Fluid Samples

Synovial fluid samples were obtained from 28 and 25 patients diagnosed with gout and rheumatoid arthritis (RA), respectively, at Asan Medical Center (Seoul, Korea). All patients had active disease at the time of sampling, for which they required joint aspiration with or without intra-articular injection of corticosteroids. Gout diagnosis was confirmed by the presence of needle-shaped MSU crystals on polarizing microscopy of the joint fluid. The diagnosis of RA was based on the 2010 revised American College of Rheumatology criteria/European League against Rheumatism criteria for the classification of RA (29). The patients’ demographic and clinical characteristics are described in Table 1. All patients provided written informed consent, and the Asan Medical Center Institutional Review Board approved the study protocol (2016-0036).

**Table 1:** Clinical characteristics of patients with gout and those with rheumatoid arthritis (RA).

	Gout (*n* = 28)	RA (*n* = 25)	*p*
Age [years, mean (SD)]	57.1 (15.4)	54.8 (14.1)	0.574
Male (%)	27 (96.4)	10 (40.0)	<0.001
CRP [mg/dl, mean (SD)]	10.5 (7.8)	2.9 (2.6)	<0.001
Uric acid [mg/dl, mean (SD)]	7.2 (2.6)	4.5 (1.6)	<0.001
Joint fluid analysis			
WBC [/μL, mean (SD)]	31,679 (25,176)	25,134 (22,358)	0.328
Neutrophil [/μL, mean (SD)]	27,774 (22,656)	18,456 (19,819)	0.122

*CRP, C-reactive protein; WBC, white blood cells*.

### Reagents and Antibodies

The following monoclonal antibodies were used for multicolor flow cytometry. Anti-CD3 (UCHT-1), anti-CD19 (HIB19), anti-CD56 (B159), anti-CD14 (MφP9), anti-CD16 (3G8), anti-CD80 (L307.4), anti-IL-1β (AS10), anti-IL-8 (6217), and anti-TNF (Mab11) were obtained from BD Bioscience (San Jose, CA, USA). Anti-CD88 (S5/1), anti-CD163 (GHI/61), anti-CD206 (15-2), anti-MRP14 (MRP1H9), and anti-IL-10 (JES3-9D7) were also used (Biolegend, San Diego, CA, USA). Anti-CX3CR1 (2A9-1), anti-IL-1 receptor antagonist (IL-1Ra; CRM17), anti-25F9 (eBio25F9), anti-MRP8 (CF-145), and Fixable Viability Dye eFluor^®^ 506 were purchased from eBioscience (San Diego, CA, USA). Anti-CCR2 (REA264) and anti-MERTK (125518) were purchased from Miltenyi Biotec (Auburn, CA, USA) and R&D Systems (Minneapolis, MN, USA), respectively. In the migration assay, anti-human C5a (R&D Systems) and/or anti-human CCL2 (R&D Systems) antibodies were used for blocking. Uric acid and lipopolysaccharide (LPS) were purchased from Sigma (St. Louis, MO, USA). RPMI1640, penicillin–streptomycin, and FBS were obtained from Gibco Inc. (Grand Island, NY, USA).

Monosodium urate crystals were prepared as described previously (30). Uric acid was dissolved in a 0.01 M NaOH solution and heated to 70°C. After the pH was adjusted between 7.1 and 7.2 using HCl, the solution was autoclaved at 121°C for 2 h. MSU crystals were harvested and resuspended in PBS (10 mg/ml). The presence of needle-shaped crystals was confirmed using polarizing light microscopy.

### Cell Isolation and Stimulation

Synovial fluid samples were harvested by centrifugation at 1,800 *g* for 10 min. Then, the pellet was collected for mononuclear cell isolation and the supernatants were stored at −80°C until chemokine measurements. Mononuclear cells were isolated from synovial fluid and peripheral blood using Ficoll-Paque™ PLUS gradient centrifugation (GE Healthcare, Piscataway, NJ, USA). Synovial fluid mononuclear cells (SFMCs) were cultured at a density of 1 × 10^6^ cells/ml in complete RPMI1640 (Gibco) containing Golgi Stop (BD Bioscience). Then, the cells were stimulated with LPS (1 µg/ml) or MSU crystals (200 µg/ml) for 5 h at 37°C.

### Flow Cytometric Analysis

To exclude dead cells from further analysis, SFMCs were incubated with Fixable Viability Dye eFluor^®^ 506 (eBioscience) for 30 min at 4°C according to the manufacturer’s instructions. This dye labels dead cells without a loss of fluorescence intensity during fixation or permeabilization step in the staining procedure (31). Cells were washed and then incubated with primary antibodies directed against surface markers or a matched isotype control for 30 min at 4°C. For intracellular cytokine staining, cells were fixed and permeabilized according to the manufacturer’s protocol (eBioscience). Cells were stained with fluorescence-conjugated antibodies against cytokine or a matched isotype control for 30 min at room temperature. After washing, samples were acquired on a BD FACSCanto™ II flow cytometer (BD Biosciences) and analyzed with FlowJo software (Tree Star, Ashland, OR, USA). Mean fluorescence intensity (MFI) was determined after subtraction of MFI using isotype control antibody.

### Cell-Migration Assay

Migration assays were performed in 5.0-µm Transwell^®^ (Costar, Corning, NY, USA) for 6 h at 37°C. Synovial fluid samples from patients with gout were diluted with media containing 1% FBS and then pre-incubated with control IgG or neutralizing antibodies (anti-C5a Ab. and anti-CCL2 Ab.) for 1 h (32). Peripheral blood mononuclear cells from healthy subjects or SFMCs from patients with gout were loaded into the upper chamber at 2 × 10^5^ cells/well and the diluted synovial fluid samples were placed in the lower chamber. After 5-h incubation, cells were harvested and counted using a flow cytometer with CountBright™ Absolute counting beads (Molecular Probes, Eugene, OR, USA). The percentage of migrated CD14^+^ monocytes was calculated relative to the total number of CD14^+^ monocytes loaded into the upper chamber. Fold change in the number of migrated cells was calculated by comparing to that of cells loaded without synovial fluid in the lower chamber.

### Analysis of MSU Crystals Phagocytosis

SFMCs were incubated with 200 µg/ml MSU crystals for 6 h at 37°C. Phagocytosis of MSU crystals was then determined by analyzing the increase of side scatter (SSc) in flow cytometer as previously described (33). MFI of the SSc was determined after subtraction of MFI using SFMCs cultured without MSU crystals.

### Statistical Analysis

Statistical analyses were performed with Prism 5 (GraphPad Software, San Diego, CA, USA). Comparisons of monocytes/macrophages or lymphocytes between groups were performed using the Mann–Whitney test. Unpaired *t*-test was used to compare the migration effect for neutralizing Ab. Correlation analyses were performed using Spearman’s rank correlation coefficients. Significance was defined as *p* value of <0.05 (*), <0.01 (**), or <0.001 (***).

## Results

### The Frequency of Monocytes/Macrophages Was Increased during an Acute Gout Attack

**Figure 1:**
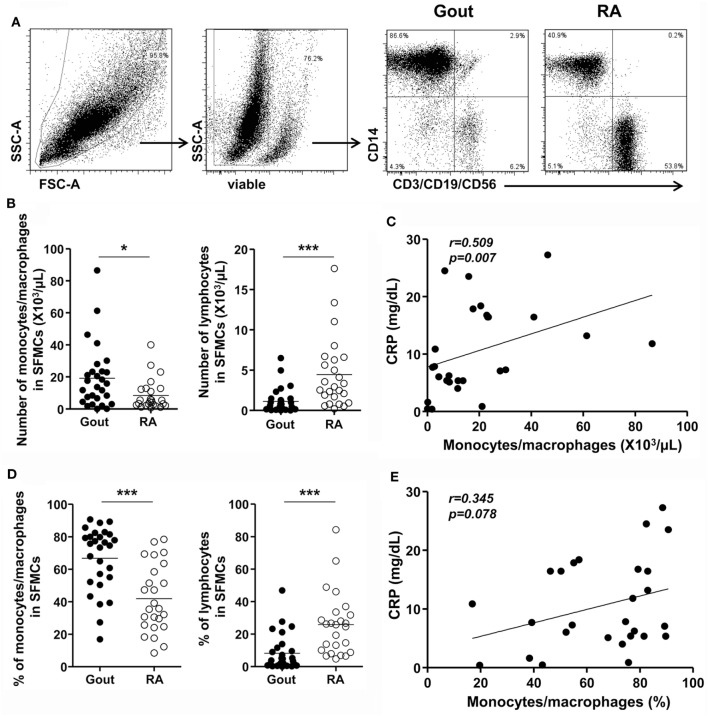
Monocytes/macrophages in the synovial fluid during an acute gout attack. **(A)** In synovial fluid mononuclear cells (SFMCs), the gating strategy for the quantification of CD14^+^CD3^−^CD19^−^CD56^−^ cells is shown. **(B,D)** The number **(B)** and frequency **(D)** of monocytes/macrophages (left) and lymphocytes (right) was determined in the SFMCs of patients with gout (*n* = 28) and rheumatoid arthritis (RA; *n* = 25). **(C,E)** The correlation between the number **(C)** and frequency **(E)** of CD14^+^ monocytes/macrophages and the levels of C-reactive protein (CRP) was analyzed in patients with gout (*n* = 27). *p: 0.05, **p: 0.01, ***p: 0.001.

We first investigated whether the number and frequency of monocytes/macrophages was increased in the synovial fluid of patients with gout, which was collected during an acute gout attack (duration after onset of acute attack: median, 2.5 days, interquartile range, 2–5 days; Figure 1A). Using multicolor flow cytometry, after the exclusion of dead cells, monocytes/macrophages were identified on the basis of the CD14 expression, but not CD3, CD19, and CD56 expression. A significant increase in the number and frequency of monocytes/macrophages was observed in patients with gout than in those with RA, which is a chronic inflammatory arthritis characterized by the activation of various inflammatory cells, including macrophages (Figures 1B,D). In contrast, the number and frequency of lymphocytes, defined as low to intermediate forward scatter and low SSc, as well as the expression of CD3, CD19, or CD56, was significantly lower in the synovial fluid of patients with gout than in that of patients with RA. In addition, we observed significant correlation between the number of monocytes/macrophages and the levels of C-reactive protein (CRP), which is indicative of an inflammatory state (Figure 1C). And although statistical significance was not reached, we found that the frequency of monocytes/macrophages was correlated with the serum levels of CRP (Figure 1E). Taken together, our results indicate that there is a significant increase and possible role of CD14^+^ monocytes/macrophages in the synovial fluid during an acute attack in patients with gout.

### CD14^+^ Cells in Gout Showed the Phenotypes of Infiltrated Monocytes

**Figure 2:**
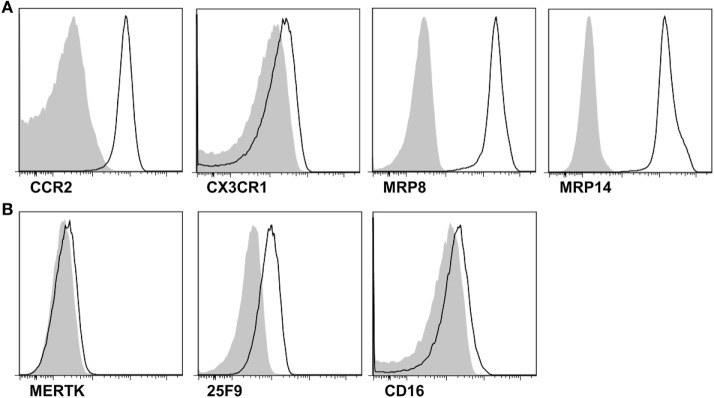
Phenotypes for infiltrated monocytes and tissue-resident macrophages in the synovial fluid. **(A,B)** In the gating of CD14^+^CD3^−^CD19^−^CD56^−^ cells, the expression of markers for infiltrated monocytes **(A)** and tissue-resident macrophages **(B)** was determined. Representative flow cytometry plots from at least five independent experiments are presented. *p: 0.05, **p: 0.01, ***p: 0.001.

Next, to address whether CD14^+^ monocytes/macrophages are recently infiltrating monocytes or tissue-resident macrophages, we examined the expression of markers for infiltrated monocytes and tissue-resident macrophages. Although negligible expression of CX3CR1 was detected, CD14^+^ monocytes/macrophages showed marked expression of the chemokine receptor CCR2, which is required for the recruitment of inflammatory monocytes to inflamed tissues (Figure 2A). Further, remarkable MRP8 and MRP14 expression was found in CD14^+^ monocytes/macrophages in gout. In contrast, MERTK and 25F9, which are present on tissue-resident macrophages, were minimally expressed on CD14^+^ cells (Figure 2B). These data demonstrated that CD14^+^ monocytes/macrophages are infiltrating monocytes, rather than tissue-resident macrophages, from the peripheral blood.

### C5a and C5a Signaling Plays an Important Role in Monocyte Migration during an Acute Gout Attack

**Figure 3:**
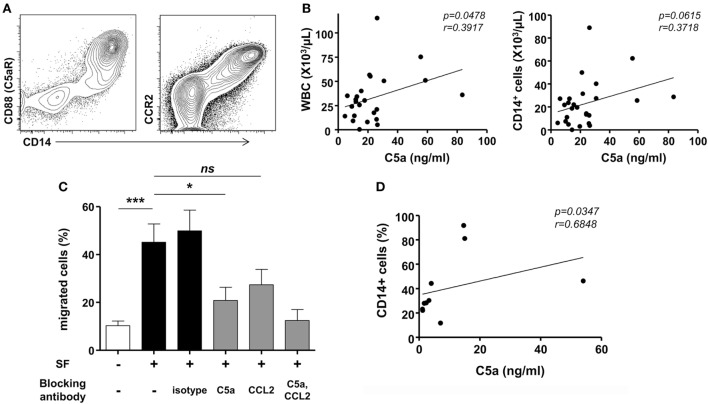
Migration of monocytes in gout. **(A)** CD88 (C5aR) and CCR2 expression was analyzed in synovial fluid mononuclear cells. Representative flow cytometry plots are presented. **(B)** Correlations between levels of C5a in synovial fluid samples and numbers of white blood cells (WBC; left) or CD14^+^ cells (right) in synovial fluid from patients with acute gout attack (*n* = 26) were analyzed. **(C)** Peripheral blood mononuclear cells in the upper chamber were co-cultured with synovial fluid samples from gout patients in the presence of anti-C5a blocking antibody, anti-CCL2 blocking antibody, or isotype antibody. Migrated CD14^+^ cells were counted with flow cytometry. **(D)** The correlation between C5a levels in synovial fluid samples and the number of migrated CD14^+^ cells to the lower chamber is presented (*n* = 10).

Further, we addressed the mechanism for monocyte infiltration into the joints during an acute attack in patients with gout. CD14^+^ cells exclusively expressed C5aR and CCR2 (Figure 3A). We then measured the levels of C5a and CCL2 in the joint fluids of patients. Although a significant difference was not detected between patients with gout and those with RA, there were significant levels of C5a (mean ± SD, 93.65 ± 352.0 ng/ml) and CCL2 (70.79 ± 99.89 ng/ml) in the joints of patients with gout. Thus, we attempted to determine the potential role of C5a and CCL2 in the migration of monocytes during an acute gout attack. There was a significant correlation between the number of infiltrated white blood cells and the level of C5a but not that of CCL2 (Figure 3B and Figure S1 in Supplementary Material). A positive correlation was observed between the number of CD14^+^ cells and C5a level in synovial fluids, although the statistical significance was low (*p* = 0.0615, *r* = 0.3718). Incubation with synovial fluids from gout patients significantly induced the migration of monocytes, and these effects were well correlated with the concentration of C5a in synovial fluid samples (Figures 3C,D). Further, neutralization of C5a but not CCL2 significantly decreased monocyte migration compared with the isotype antibody-treated group. Then, assays were performed using synovial fluid cells from patients with gout. Although the statistical significance was not reached (*p* = 0.064), the incubation of synovial fluid cells from gout patients in matched synovial fluids caused the migration of CD14^+^ monocytes/macrophages toward the fluid (Figure S2A in Supplementary Material). Also, there was a trend of inhibition of CD14^+^ cells migration after blockade with anti-C5a neutralizing antibody. We observed significant correlation between C5a concentration in synovial fluid and the percentage of migrated CD14^+^ cells among SFMCs loaded in the upper compartment (Figure S2B in Supplementary Material). Given that C5a concentration in joint fluid obtained from gout patients was diverse among individuals, these results were analyzed by fold change relative to media control in the lower chamber. The results showed that there was a significant inhibition of CD14^+^ cells migration after neutralization of C5a, but not of CCL2 (Figure S2C in Supplementary Material). These results suggested that C5a signaling plays an important role in monocyte migration into the joints of gout patients during an acute attack.

### Monocytes Had Anti-inflammatory as well as Pro-inflammatory Features in Gout

**Figure 4:**
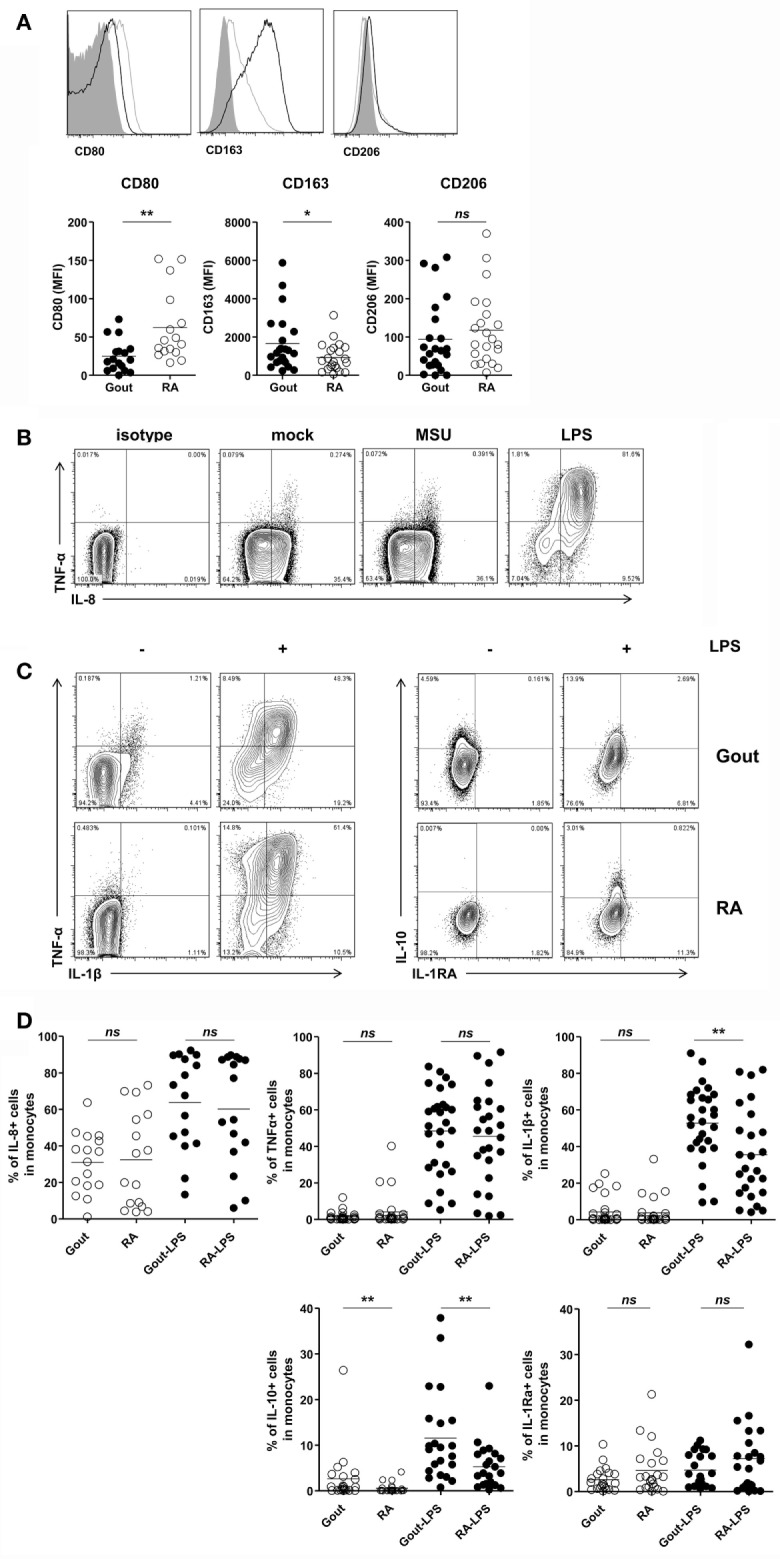
Pro-inflammatory and anti-inflammatory phenotypes of CD14^+^ monocytes/macrophages in patients with gout. **(A)** CD80, CD163, and CD206 expression was determined and analyzed in CD14^+^ cells from patients with gout (*n* = 23, except for CD80 where *n* = 17) and those with rheumatoid arthritis (RA) (*n* = 22, except for CD80 where *n* = 16). MFI, mean fluorescence intensity. **(B)** From within the gate of CD14^+^CD3^−^CD19^−^CD56^−^ cells, TNF-α and IL-8 expression was determined after stimulation with or without monosodium urate (MSU) or lipopolysaccharide (LPS). Representative flow cytometry plots are presented. **(C)** From within the gate of CD14^+^CD3^−^CD19^−^CD56^−^ cells, TNF-α, IL-1β (left), and IL-10, IL-1Ra (right) production in patients with gout or RA was analyzed after stimulation with or without LPS. Representative flow cytometry plots are presented. **(D)** A comparison between patients with gout and RA is presented for the production of IL-8 (*n* = 16 for gout; *n* = 16 for RA), TNF-α (*n* = 28 for gout; *n* = 25 for RA), IL-1β (*n* = 28 for gout, *n* = 25 for RA), IL-10 (*n* = 22 for gout; *n* = 22 for RA), and IL-1Ra (*n* = 22 for gout, *n* = 22 for RA) in CD14^+^CD3^−^CD19^−^CD56^−^ cells. *p: 0.05, **p: 0.01, ***p: 0.001.

Then, we evaluated the inflammatory phenotypes of CD14^+^ monocytes/macrophages in patients with gout. We found a significantly lower expression of CD80, one of the markers of M1 macrophages, in gout patients than in RA patients (Figure 4A). In contrast, CD14^+^ cells in gout patients showed marked expression of CD163, a marker for alternative activation of macrophages (M2). There was no significant difference in the CD206 expression between gout and RA patients. We also examined cytokine production from the monocytes/macrophages of patients with gout. Intracellular cytokine staining showed considerable amounts of IL-8 from CD14^+^ cells even without re-stimulation (Figure 4B). However, a significant increase of pro-inflammatory cytokines, including TNF-α and IL-8, was not detected after MSU re-stimulation. Notably, after stimulation with LPS, substantial amounts of IL-1β, IL-8, and TNF-α were detected in patients with gout (Figure 4C). CD14^+^ cells from gout patients produced significantly higher levels of IL-1β secretion compared to cells from RA patients. In addition, the production of anti-inflammatory cytokines (IL-10 and IL-1Ra) was examined in patients with or without stimulation with LPS. We found that IL-10 production from monocytes/macrophages of gout patients was significantly higher compared to the production in RA patients (Figures 4C,D). Taken together, these data suggest that CD14^+^ cells from patients with gout are infiltrated monocytes and exhibit phenotypes of anti-inflammatory as well as pro-inflammatory characteristics.

### CD14^+^ Cells Expressing M2 Phenotype Had Phagocytic Activity for MSU Crystals in Gout

**Figure 5:**
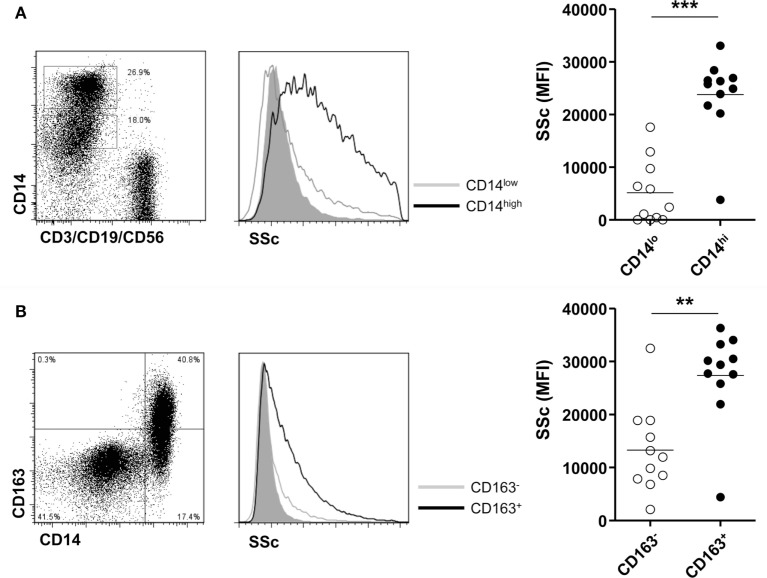
Phagocytosis for monosodium urate (MSU) crystals of CD14^+^ monocytes/macrophages in patients with gout. **(A)** Side scatter (SSc) was determined and analyzed between CD14 high-expressing and CD14 low-expressing cells among CD3^−^CD19^−^CD56^−^ cells of synovial fluid mononuclear cells from patients with gout (*n* = 11). **(B)** A comparison of SSc values between CD163^+^ CD14^+^ cells and CD163^−^ CD14^+^ cells after incubation with MSU crystals (*n* = 11) was analyzed by flow cytometry.

Finally, we investigated the phagocytic activity of CD14^+^ monocytes/macrophages in patients with gout. There was a significant difference in phagocytosis of MSU crystals between CD14 high-expressing and CD14 low-expressing cells among monocytes/macrophages (Figure 5A). Then, to determine whether M2 macrophages have a higher phagocytic activity, we compared the phagocytosis of CD163^+^ CD14^+^ cells with that of CD163^-^ CD14^+^ cells after incubation with MSU crystals. We found a significantly higher increase of SSc in CD163^+^ than in CD163^−^ monocytes/macrophages (Figure 5B). It indicated that CD14^+^ cells with phenotypes of M2 in gouty arthritis have higher phagocytic activity for MSU crystals.

## Discussion

In the present study, we demonstrated that the number and frequency of CD14^+^ monocytes/macrophages was significantly increased in the joint fluids of gout patients during an acute gout attack. These CD14^+^ cells exhibited the phenotype of infiltrating monocytes rather than that of tissue-resident macrophages. Further, we found that C5a levels in the joints were correlated with monocyte migration, and synovial fluids from gout patients induced monocyte migration, which was significantly decreased after blocking with anti-C5a antibody. In addition, monocytes from gout patients exhibited anti-inflammatory as well as pro-inflammatory activity, demonstrated by the production of IL-1β, IL-8, TNF-α, and IL-10.

It has been suggested that monocyte/macrophage lineage cells are initial responders in the initiation of gouty inflammation. Synovial cells can reportedly produce inflammatory cytokines such as TNF-α and IL-6 in response to MSU crystals (3, 34). Further, the phenotypes and functional characteristics of monocytes/macrophages have been described in a murine MSU-induced peritonitis model (6, 28). This report demonstrated that tissue-resident macrophages are key cells for the initiation of neutrophilic inflammation through the production of IL-1β and TNFα in an MSU crystal-induced inflammation model in rodents. In the present study, we also found increased amounts of CD14^+^ monocytes/macrophages in the synovial fluid samples of patients with acute gout attacks. The number of these cells was correlated with inflammatory activity, as assessed by CRP levels (Figure 1C). These results suggested that monocytes/macrophages are implicated in the pathogenesis of acute inflammation in gouty arthritis. Our study, in contrast with previous data obtained from MSU-mediated inflammation in a murine model, indicated that the majority of CD14^+^ cells exhibited a phenotype of infiltrated monocytes but not that of tissue-resident macrophages, evidenced by high CCR2, MRP8, and MRP14 expression with negligible MERTK and 25F9 expression (Figure 2). Although we cannot fully address the kinetic change of the monocyte/macrophage population during the disease course of a gout attack, a consistent pattern of infiltrated monocytes was observed in all synovial cells obtained from patients with different times of gout attack onset (data not shown). These findings indicated that there is a significant recruitment of monocytes during an acute gout attack. Further studies are required to elucidate the fate and role of infiltrated monocytes in determining the clinical manifestations of gout, including the spontaneous resolution of acute inflammation.

The recruitment of monocytes from peripheral blood into the tissues contributes to the development of many inflammatory diseases as well as the control of infections (12, 13). Trafficking of monocytes to the site of inflammation or infection requires coordinated action by various chemoattractant molecules, including CCL2, which are released by inflamed tissue. Indeed, previous study have shown that elevated CCL2 levels in the serum are correlated with increased amounts of circulating CD14^+^ monocytes in subjects with gout and asymptomatic hyperuricemia, suggesting the possible role of CCL2 in the priming and trafficking of monocytes in gout (35). However, interestingly, our present results showed that the levels of C5a but not CCL2 were correlated with the number of leukocytes in the joints (Figure 3B and Figure S1 in Supplementary Material). Further, blocking of C5a but not of CCL2 significantly decreased the monocyte migration in response to joint fluids from gout patients (Figure 3C and Figure S2 in Supplementary Material). These findings indicated that C5a signaling plays an important role in monocyte infiltration during an acute attack in patients with gout.

In the present study, IL-8 was readily produced by monocytes without stimulation (Figure 4B). It is well known that IL-8 is one of the key chemokines involved in neutrophil migration (36). In addition, blockade of IL-8 attenuated joint swelling and neutrophil infiltration after MSU injection in rabbits (37). Thus, given that robust and rapid neutrophil infiltration is a characteristic feature in gouty arthritis, IL-8 production from monocytes might be responsible for neutrophilic inflammation in gout.

One of the most interesting features in gouty inflammation is that despite marked and severe manifestation in the onset of clinical presentation, the condition is spontaneously resolved without treatment. Several mechanisms have been proposed to explain the resolution of acute gouty inflammation (38). In the present study, we examined anti-inflammatory activity according to the expression of M2 markers and anti-inflammatory cytokines, and found increased CD163 expression (Figure 4A). Further, significantly higher levels of IL-10 were detected with or without stimulation in CD14^+^ cells from patients with gout compared to those with RA (Figure 4C). CD163-expressing macrophages exhibited a significantly higher phagocytic activity for MSU crystals (Figure 5) than CD163-negative macrophages. Interestingly, the acquisition of the anti-inflammatory phenotype with CD163 and TGF-β expression from differentiated macrophages after stimulation with MSU crystals has been reported (21, 39). In view of the recent research findings, subsets of macrophages can share both pro-inflammatory M1 and anti-inflammatory M2 characteristics rather than a distinct population (40). Our present results also showed the significant production of pro-inflammatory cytokines, including IL-1β and TNF-α, in monocytes from patients with gout. Thus, it is appealing to hypothesize that a diverse response to MSU crystals, including M1 and M2 heterogeneity, can contribute to the dynamic clinical presentation and spontaneous resolution of gout.

In conclusion, we demonstrated that the number and frequency of CD14^+^CD3^−^CD19^−^CD56^−^ monocytes/macrophages was markedly increased in the synovial fluid cells of patients with gout. CD14^+^ cells showed the phenotypes of infiltrated monocytes rather than tissue-resident macrophages characterized by high CCR2, MRP8, and MRP14 expression but low MERTK and 25F9 expression. These cells displayed increased M2 markers such as CD163, and had the capacity to produce TNF-α, IL-1β, and IL-10. Moreover, CD14^+^ cells with phenotype of M2 macrophages had high phagocytic activity for MSU crystals. Taken together, these data suggest that monocytes/macrophages from patients with acute gout attack are infiltrated monocytes, and exhibit anti-inflammatory as well as pro-inflammatory characteristics, possibly contributing to the robust but spontaneous resolution of gouty inflammation.

## Ethics Statement

This study was approved by the Asan Medical Center Institutional Review Board (2016-0036).

## Author Contributions

JJ, SH, and Y-GK designed the study. JJ, IH, and SH performed the experiments. JJ, SH, OK, BG, Y-GK, C-KL, and BY analyzed the data. SH, Y-GK, C-KL, and BY provided the clinical samples. JJ and SH wrote the manuscripts.

## Conflict of Interest Statement

The authors declare that the research was conducted in the absence of any commercial or financial relationships that could be construed as a potential conflict of interest.

## Supplementary Material

Figure S1 Correlation between the concentration of CCL2 and leukocyte migration in gout. Correlation analysis of CCL2 levels with numbers of white blood cells (left) or CD14^+^ cells in synovial fluid in gout patients.

Figure S2 Migration of CD14^+^ cells in synovial fluid mononuclear cells (SFMCs) from patients with gout. SFMCs in the upper chamber were co-cultured with synovial fluid samples from gout patients in the presence of either anti-C5a blocking antibody, anti-CCL2 blocking antibody, or isotype antibody in the lower chamber. **(A)** Numbers of migrated CD14^+^ cells in the lower chamber were counted with flow cytometry (*n* = 9). **(B)** The correlation between C5a levels in synovial fluid and the number of migrated CD14^+^ cells among SFMCs is presented (*n* = 9). **(C)** Fold change relative to media control in the lower chamber is shown (*n* = 9).
