# Supplementary figures and images for: CD14+ Cells with the Phenotype of Infiltrated Monocytes Consist of Distinct Populations Characterized by Anti-inflammatory as well as Pro-inflammatory Activity in Gouty Arthritis
Source: Front Immunol. 2017 Oct 6;8:1260. doi: 10.3389/fimmu.2017.01260 (PMC5635328; doi:10.3389/fimmu.2017.01260)

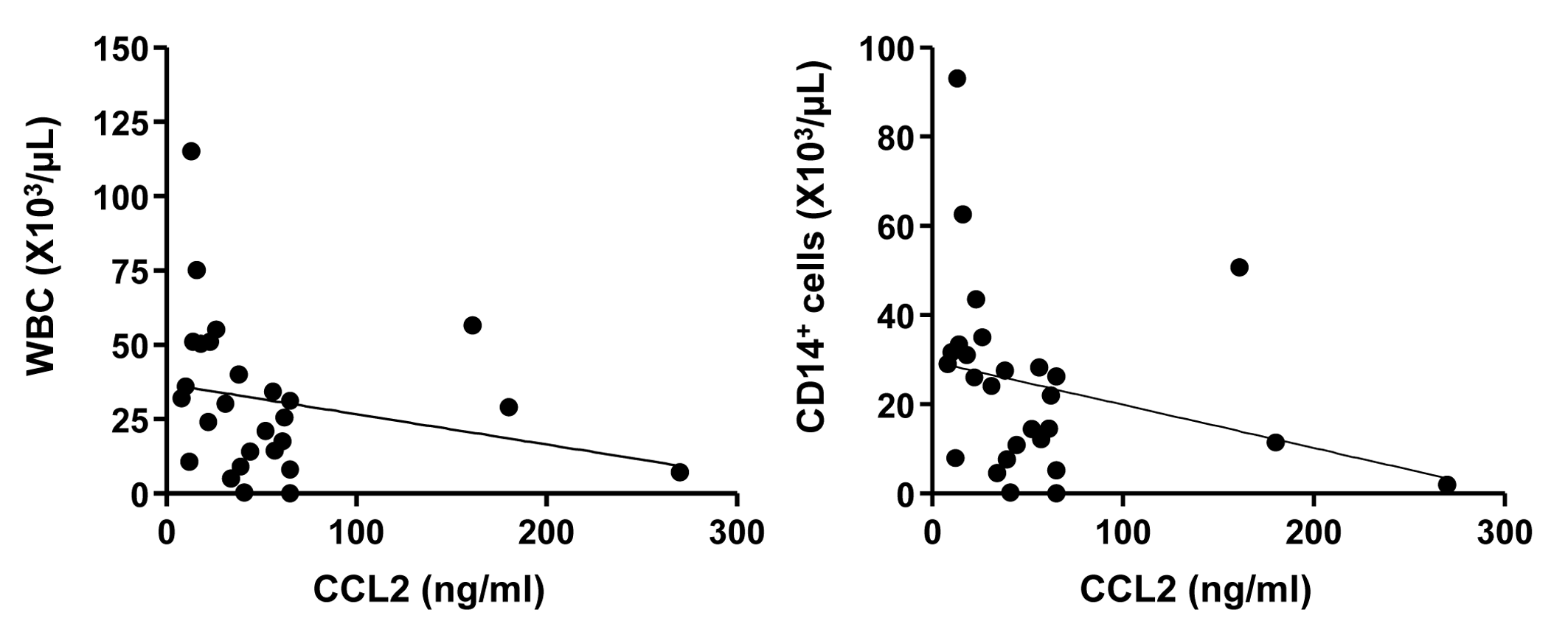

Supplement: Figure S1 — Correlation between the concentration of CCL2 and leukocyte migration in gout. Correlation analysis of CCL2 levels with numbers of white blood cells (left) or CD14+ cells in synovial fluid in gout patients. [file Image_1.TIF]

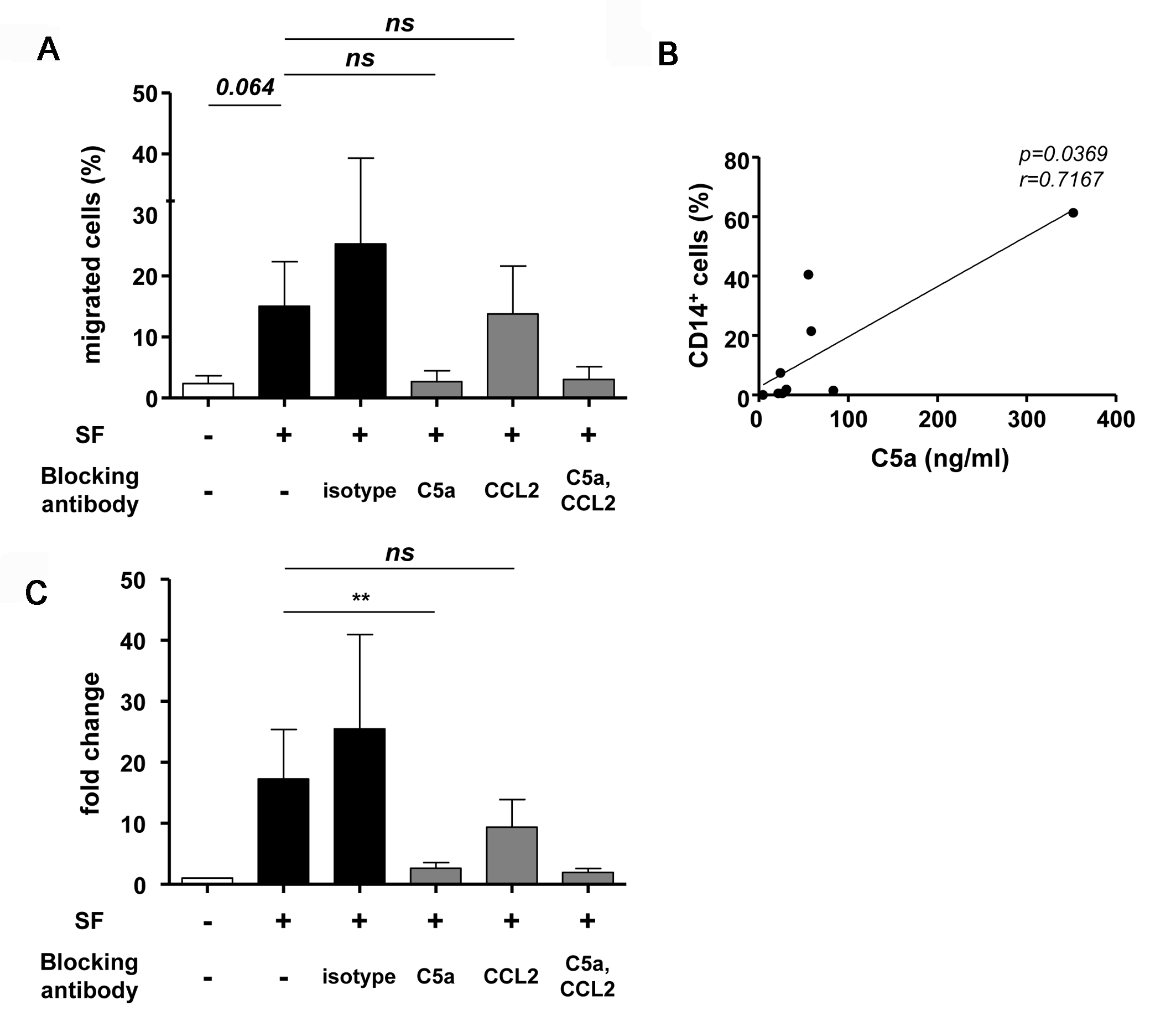

Supplement: Figure S2 — Migration of CD14+ cells in synovial fluid mononuclear cells (SFMCs) from patients with gout. SFMCs in the upper chamber were co-cultured with synovial fluid samples from gout patients in the presence of either anti-C5a blocking antibody, anti-CCL2 blocking antibody, or isotype antibody in the lower chamber. (A) Numbers of migrated CD14+ cells in the lower chamber were counted with flow cytometry (n = 9). (B) The correlation between C5a levels in synovial fluid and the number of migrated CD14+ cells among SFMCs is presented (n = 9). (C) Fold change relative to media control in the lower chamber is shown (n = 9). [file Image_2.TIF]
